# Sexualized drug use among men who have sex with men in Madrid and Barcelona: The gateway to new drug use?

**DOI:** 10.3389/fpubh.2022.997730

**Published:** 2022-11-15

**Authors:** Juan-Miguel Guerras, Juan Hoyos, Marta Donat, Luis de la Fuente, David Palma Díaz, Oskar Ayerdi, Jorge N. García-Pérez, Patricia García de Olalla, María-José Belza

**Affiliations:** ^1^Escuela Nacional de Sanidad, Instituto de Salud Carlos III, Madrid, Spain; ^2^CIBER Epidemiologia y Salud Pública (CIBERESP), Madrid, Spain; ^3^Independent Researcher, Madrid, Spain; ^4^Centro Nacional de Epidemiología, Instituto de Salud Carlos III, Madrid, Spain; ^5^Servicio de Epidemiología, Agència de Salut Pública de Barcelona, Barcelona, Spain; ^6^Centro Sanitario Sandoval, Instituto de Investigación Sanitaria San Carlos, Hospital Clínico San Carlos, Madrid, Spain; ^7^Unidad de ITS de Vall d'Hebron-Drassanes, Hospital Vall d'Hebron, Barcelona, Spain; ^8^Institut d'Investigació Biomèdica Sant Pau, Hospital Universitari Sant Pau, Barcelona, Spain

**Keywords:** men who have sex with men, sexualized drug use, chemsex, drug use, drug initiation

## Abstract

This original study compares the prevalences of drug use for any purpose and for sexualized drug use (SDU) among MSM. It also describes relevant characteristics of first SDU, analyzes to what extent SDU has been the first experience (the gateway) with different drugs by age and explores the correlates of SDU. Study participants included 2,919 HIV-negative MSM attending four HIV/STI diagnosis services in Madrid and Barcelona. They answered an online, self-administered questionnaire. Poisson regression models with robust variance were used. About 81.4% had ever used any drug, and 71.9% had done so in the last-12-months, while 56% had ever engaged in SDU, and 50% had done so in the last-12-months. Participants under 25 years old had the lowest prevalences of SDU, and the 25–39 age group the highest, except for Viagra, which was higher among those over age 40. The most frequently used drugs for first SDU were poppers (53.6%), cannabis (19.6%) and Viagra (12.2%). These drugs were also the most ever consumed for SDU. Among sexualized users, methamphetamine (78.3%) and Mephedrone (75.4%) were used always/most of the times for sex in the last-12-months. Around 72.2% of Mephedrone sexualized users and 69.6% of Methamphetamine vs 23.1% of ecstasy users' first consumption of these drugs involved use for sex. These drugs were provided to them free where they have sex for 66.8, 79.1, and 31.9%, respectively. On that occasion, 8.1% of Mephedrone, 6.8% of Methamphetamine and 18.4% of ecstasy users had sex only with steady partner; with 50.2, 56.2, and 26.2% respectively using a condom with any partner. SDU in the first use was associated with similar variables for recreational and chemsex drugs. The highest prevalence ratios were for having ever been penetrated by >20 men and having ever injected drugs. It can be concluded that the prevalence of SDU was more than half of the prevalence for any purpose. Thus SDU was the gateway to use for many drugs in an important proportion of users, who frequently consumed drugs that were free and had condomless anal sex with occasional and multiple partners. These circumstances were much more common for chemsex than for recreational drugs.

## Introduction

In the last decade, much research has been published on Sexualized Drug Use (SDU) by men who have sex with men (MSM) (1–4) which involves drug use just before or during sex. Most of these studies have focused on what in the UK is known as “chemsex,” which involves sexual encounters between MSM (usually group-sex) under the effects of certain drugs. Chemsex has recently become widespread among MSM. The most commonly labeled “chemsex drugs,” or “4-chem” drugs include (5): methamphetamine, mephedrone, GHB/GBL, and ketamine. “3-chem” refers to methamphetamine, mephedrone, GHB/GBL (1, 6, 7), and “2-chem” refers to the two most closely related drugs: methamphetamine and mephedrone. The growth of this phenomenon has been closely linked to the proliferation of geosocial networking dating apps which facilitate both contact with potential sexual partners and the acquisition of substances (8). Geosocial networking dating apps facilitate and multiply the possibilities of finding sexual partners with whom to engage in these practices, and can play a relevant role in the practice of chemsex (9). The public health significance of SDU is primarily related to the fact that chemsex drugs have been associated with high risk sexual behaviors and an increased likelihood of acquiring HIV, HCV and other STIs (4, 6). Individuals who use these substances are on the top of the “risk ladder,” according to the type of drugs used for SDU, especially among those who practice chemsex (10), being a priority group to be included in PrEP programs (11). Other health consequences, such as substance dependence (12), mental health effects (13, 14) and deaths from overdose- in particular by GHB (15, 16) or methamphetamine (12)- have been much less studied, but should not be underestimated.

Although the chemsex phenomenon was first described in the United Kingdom and has been investigated mainly in Western countries, it is a behavior that has been observed internationally (1–4). Many studies only consider three or four chemsex drugs (5, 14, 17). The label “chemsex” is frequently used, when in reality, studies should use the term SDU, as they include substances other than chemsex drugs (18–21). Many studies have focused on assessing whether or not users of particular drugs have a higher prevalence of risky sexual behaviors or a higher incidence of HIV, but it is not always possible to discriminate SDU from recreational drug use. Although, in many cases, SDU is probably the appropriate term (22–24), especially for certain substances, in other cases we lack information in this regard (25–31). One of the most studied substances, especially in the USA, is methamphetamine (23, 24, 29, 31–33), which, together with mephedrone, is one of the drugs most associated with chemsex.

Studies of a wide range of drugs show that the prevalence of SDU of the most commonly used drugs for any purpose (such as cannabis or cocaine) tend to be much higher than the prevalence of chemsex drugs (10, 34). Besides making the sexual experience more intense, pleasurable or long-lasting, there are many other reasons for drug use (recreational, performance enhancement, etc.), Analysis of the available information suggests that, with the exception of poppers and the 4-chems, psychoactive drugs are probably used more frequently by most MSM for purposes other than sex. However, there are no studies comparing the prevalence of sexualized use of a broad range of drugs with the prevalence of use for any purpose. Likewise, we can assume that SDU (whether one has taken the substance intentionally for sex or not), could be the first experience (the gateway) with different drugs for a significant percentage of users, and it is possible that it serves as an exclusive gateway for chemsex drugs. However, there are no studies that confirm or refute these hypothesis. Likewise, there is no additional drug-specific information on the circumstances surrounding the first sexualized use, as almost all studies focus on the last SDU or the last sexual encounter (34, 35).

This study focuses on MSM and aims to estimate the prevalence of use of certain drugs for any purpose and specifically for sex, the percentage of participants for whom each substance was the first drug or the most commonly used drug for sex, and the proportion who used the drug always or most of the time for sex. We also estimate, the proportion of participants for whom SDU was the gateway to use by drug and age group. We also describe some relevant characteristics of the first SDU by drug.

## Methods

### Project design and sample recruitment

The Methysos Project is a research project that aims to analyze the prevalence and characteristics of drug use for any purpose, with a special focus on SDU, in MSM living in Spain. The project was funded by the National Plan on Drugs and was approved by the Research Ethics Committee of the Instituto de Salud Carlos III (CEI PI 44_2018_subproyecto1-v2 and CEI PI 44_2018_subproyecto2). In this study, we present the results from the first survey of the project, carried out in four diagnostic facilities in the two largest Spanish cities: the two most important sexually transmitted infection clinics in Spain—Sandoval, in Madrid and Drassanes, in Barcelona—and in two community programs for rapid HIV-testing-the Pink Peace Program, in Madrid, and the Public Health Agency, in Barcelona-. The STI clinics provide on-demand services (including Prep) and perform laboratory based testing for all STIs, whereas the community programs also carry out different kinds of active recruitment (including ads and profiles on dating apps for MSM), and only offer rapid testing for HIV, syphilis and sometimes for HCV.

The study included only MSM without a previous HIV diagnosis, because this group accounted for the vast majority of attendees, and we preferred to obtain a homogenous group to increase the external validity and comparability of the results. Therefore, of all those MSM who accessed these four facilities, only those whose last HIV test had been negative or those who had never had a HIV test were offered the opportunity to participate (Supplementary Figure 1 of Annex). Those who accepted signed an informed consent. The recruitment of the sample included in this study took place from September 2018 to December 2020.

No sample size calculation was performed. We attempted to recruit a sufficiently large number of participants using non-probabilistic convenience sampling to have adequate statistical power to accurately estimate outcomes and detect statistically significant differences between subgroups of interest.

The final sample of the study was 2,919 participants: 1,816 from Madrid (627 from the STI center and 1,189 from the community center) and 1,103 from Barcelona (421 from the STI center and 682 from the community center).

### Data collection instruments

Participants answered a self-administered online questionnaire, without personal identifiers, on a tablet, while waiting to be seen. The questionnaire included sections on sociodemographics, sexual behavior, sexual and injecting risky behaviors, history of HIV and other STI testing, and drug use for any purpose and for sexualized drug use. To simplify the reading of this article and offer the opportunity for an in depth look at the contents of the questionnaire, Supplementary Table 1 of the Annex presents all the variables addressed in this article and their corresponding original categories.

**Figure 1:**
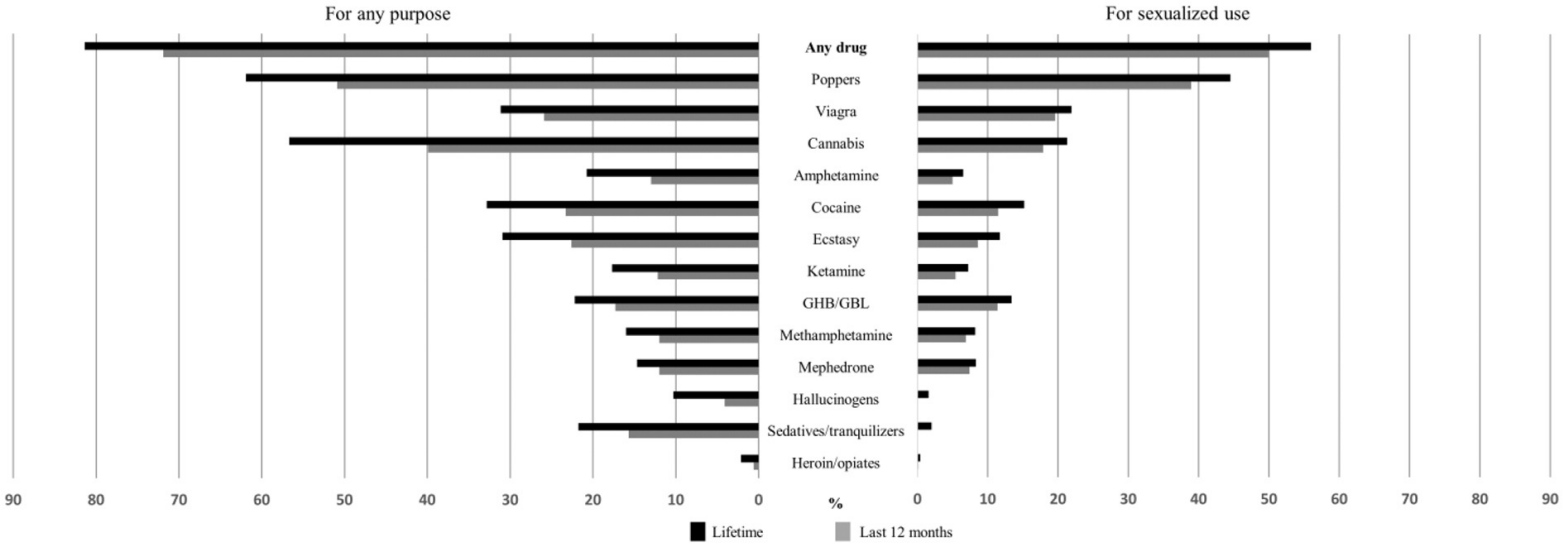
Lifetime and last 12 months prevalence of drug use for any purpose and for sexualized drug use, among MSM* from Madrid and Barcelona (%). *MSM, men who have sex with men.

In the drug use module, we first inquired about the use of drugs for any purpose: “When was the last time you used any of the following drugs?” Table 1 and Figure 1 show the 13 substances or groups of substances that we asked about. We included sex performance enhancing drugs like Viagra or similar drugs, although these substances are not psychoactive. We next inquired about SDU: “Which drugs have you ever consumed during anal sex or in the previous 6 h?” Then, we inquired about the first two drugs the individual had used for sex, and which were the two drugs most used for sex in his lifetime. In order to keep the questionnaire brief and to ensure a better questionnaire completion, several supplementary questions were asked pertaining to certain drugs. No further questions were proposed for hallucinogens, tranquilizers, and opiates, due to the very low expected prevalence. For the rest of the drugs, two more questions were asked: “when was the last time you used this substance to have sex?” and, “in the last 12 months, when you have used this substance, has it been: (1) always just before or during sex; (2) most of the time just before or during sex, (3) half of the time just before or during sex; (4) few times for sex.” Except for poppers, Viagra and cannabis, we also proposed a set of questions related to the first time each substance was used for sex: (1) whether the first time he used for sex was the first time he used for any purpose, and if not, (2) how many days had he used it before; (3) if it was used intentionally for sex; (4) and with which types of partners he had sex on that occasion and with which partners he had used condoms.

**Table 1:** Sample characteristics of MSM^*^ from Madrid and Barcelona by lifetime sexualized drug use (*N* = 2,919).

	**Ever sexualized drug use**		**Never sexualized drug use**		**Total**		***P*** **-value**
	***N*** = **1,636**		***N*** = **1,283**		***N*** = **2,919**		
	***N***	**%**	***N***	**%**	***N***	**%**	
**Recruitment**							
**City of testing**							0.927
Madrid	1,019	62.3	797	62.1	1,816	62.2	
Barcelona	617	37.7	486	37.9	1,103	37.8	
**Kind of testing program**							<0.001
Comunity program	1,005	61.4	866	67.5	1,871	64.1	
STI diagnostic center	631	38.6	417	32.5	1,048	35.9	
**Sociodemographics**							
**Age (years)**							<0.001
<25	218	13.3	234	18.2	452	15.5	
25–39	985	60.2	694	54.1	1,679	57.5	
≥40	433	26.5	355	27.7	788	27.0	
**Country of birth**							0.427
Spain	975	59.6	794	61.9	1,769	60.6	
Latin America	475	29.0	356	27.7	831	28.5	
Others	186	11.4	133	10.4	319	10.9	
**Size of city of residence (last 12 months)**							0.365
≤100.000	162	10.0	148	11.6	310	10.7	
100.000–1 million	197	12.2	157	12.3	354	12.2	
>1 million	1,262	77.9	971	76.1	2,233	77.1	
**Level of education**							0.986
Up to upper secondary	109	6.7	85	6.7	194	6.7	
Post-secondary	553	33.9	437	34.2	990	34.1	
University	968	59.4	755	59.1	1,723	59.3	
Employment status (last 12 months)^**^							<0.001
Employed	772	76.8	578	72.8	1,350	75.0	
Unemployed	85	8.5	47	5.9	132	7.3	
Others	148	14.7	169	21.3	317	17.6	
**Economic situation (last 12 months)**							0.267
Comfortable/It is OK	964	59.5	777	60.7	1,741	60.0	
Tight	522	32.2	383	29.9	905	31.2	
Dificult/very difficult	133	8.2	121	9.4	254	8.8	
Co-habitation (last 12 months)^**^							0.028
Alone	383	38.0	343	43.1	726	40.3	
With some people	624	62.0	452	56.9	1,076	59.7	
**Sexual behavior**							
**Gender of sex partners (ever)**							0.015
Only men	990	60.5	833	64.9	1,823	62.5	
Men & women	646	39.5	450	35.1	1,096	37.5	
**Age at first sexual intercourse with another men (years)**							<0.001
≤15	314	19.2	183	14.3	497	17.0	
16–20	934	57.1	663	51.8	1,597	54.7	
21–24	237	14.5	224	17.5	461	15.8	
≥25	151	9.2	211	16.5	362	12.4	
**Lives sex life with men**							<0.001
Openly	1,075	66.5	679	53.0	1,754	60.6	
Not openly	541	33.5	601	47.0	1,142	39.4	
**Place where the largest number of partner were found**							<0.001
Dicos/clubs/bars	258	16.2	137	11.2	395	14.0	
Saunas	188	11.8	97	7.9	285	10.1	
Private parties	91	5.7	13	1.1	104	3.7	
Apps/webs	979	61.3	867	70.6	1,846	65.3	
Cruising places	48	3.0	48	3.9	96	3.4	
Others/no search	33	2.1	66	5.4	99	3.5	
**Place where the largest number of partner were found**							<0.001
Discos/bars-saunas-private parties	537	33.6	247	20.1	784	27.8	
Others	1,060	66.4	981	79.9	2,041	72.3	
**Risk behavior**							
**Number of men who had penetrated you (ever)**							<0.001
None-one	79	4.8	223	17.4	302	10.4	
2–20	686	41.9	698	54.4	1,384	47.4	
>20	871	53.2	362	28.2	1,233	42.2	
**Number of men who had penetrated you (last 12 months)**							<0.001
None-one	461	28.2	651	50.9	1,112	38.1	
1–5	462	28.2	359	28.1	821	28.2	
>5	713	43.6	270	21.1	983	33.7	
**Ever been paid for sex**							<0.001
No	1,173	71.7	1,091	85.0	2,264	77.6	
Yes	462	28.3	192	15.0	654	22.4	
**Ever paid for sex**							<0.001
No	1,304	79.7	1,095	85.3	2,399	82.2	
Yes	332	20.3	188	14.7	520	17.8	
**Ever injected drugs**							<0.001
No	1,557	96.1	1,277	99.6	2,834	97.6	
Yes	64	3.9	5	0.4	69	2.4	
**Ever injected steroids**							<0.001
No	1,493	91.9	1,242	96.8	2,735	94.1	
Yes	131	8.1	41	3.2	172	5.9	
**History of HIV and other STI testing**							
**Time since last HIV test**							<0.001
<6 months	875	53.5	528	41.2	1,403	48.1	
>6 months	703	43.0	633	49.4	1,336	45.8	
Never tested before	57	3.5	120	9.4	177	6.1	
**HIV diagnosis in the recruitment consultation**							0.953
No	1,585	98.1	1,247	98.1	2,832	98.1	
Yes	31	1.9	24	1.9	55	1.9	
**Ever diagnosed with an STI**							<0.001
No	533	32.7	714	56.5	1,247	43.1	
Yes	1,097	67.3	550	43.5	1,647	56.9	

^*^MSM, men who have sex with men.

** These questions were not included in Barcelona.

Detailed definitions of the drug use variables in the questionnaire, as well as a summary of the terms used to group the drugs can be found in Supplementary Tables 2, 3 of the Annex.

### Statistical analysis

Most of the variables were collected in a more disaggregated form than presented here, since some of the original categories were grouped together based on their frequencies and the rationale for the analysis. Supplementary Table 1 of the Annex shows how the original variables and categories from the questionnaire were managed to obtain the final variables and categories used. The tables and figures in this article present the considered substances in a different order than that which was used in the questionnaire, to facilitate understanding. Those with similar results have been grouped together.

Comparisons of independent variables were assessed using Pearson's χ^2^ and Fisher's exact tests. For the analysis of correlates of sexualized use for the first use of recreational drugs and of chemsex drugs, Poisson regression models with robust variance were used (36). Both crude and adjusted prevalence ratios (cPRs and aPRs) and 95% confidence intervals (95%CI) were calculated. Variables with a significance level of <0.25 in the bivariate analysis were introduced into the multivariate model—after collapsing the number of categories for some variables. The Akaike Information Criteria was used to perform model comparisons in order to select the final model.

These analyzes were performed using Stata v. 17 (Statacorp, College Station, TX).

## Results

### General characteristics of the sample

In terms of sociodemographic characteristics: 73.0% of the participants were under 40 years of age, 39.4% were born abroad, 77.1% lived in the municipalities of Madrid or Barcelona, 59.3% had university-level studies, 60.0% had a comfortable economic situation and 40.3% had lived alone during the last 12 months. Concerning sexual and risk behavior: 62.5% had only ever had sex with men, 17.0% had had their first sexual relationship with a man before age 16, 60.6% lived their sexual life with men openly, 65.3% had met most of their partners through websites or dating apps; 5.5% had never been penetrated and 42.2.% had been penetrated by more than 20 men in their lives; 22.4% had been paid for sex; 17.8% had paid for sex; 2.4% had ever injected drugs and 5.9% had injected steroids. Regarding HIV and STI testing, 48.1% had been tested for HIV in the last 6 months and just 6.1% had never been tested before. About 1.9% were diagnosed with an HIV infection in that consultation, and 56.9% had at some point been diagnosed with an STI (Table 1).

### Prevalence of drug use for any purpose and of sexualized drug use

As shown in Figure 1, some 81.4% of participants had ever used any drug, and 71.9% had done so in the last 12 months. Except for opiates and hallucinogens, all substances had been used by more than 10% in the last 12 months. However, some substances had very high prevalences of use: 61.9% of the participants had ever used poppers and 56.7% cannabis; 50.9% and 39.9%, respectively, had done so in the last 12 months. In terms of sexualized use, 56.0% had ever had sexualized drug use and 49.9% had done so in the last 12 months. Opiates, sedatives and hallucinogens had ever been used by 2% or less of participants; amphetamines, ketamine, methamphetamine and mephedrone had been used by between 6.5% and 8.5 %, cannabis and Viagra by 21% and poppers by 44.5%. Prevalences for the last 12 months were similar, but a little lower (between 2 and 5 percentage points).

### Characteristics of sexualized drug use of different substances by age

Table 2 shows that the <25 age group had significantly lower prevalences of SDU for most of the drugs than older age groups. The 25–39 age group had the highest prevalences, except for Viagra which was significantly higher among those over age 40.

**Table 2:** Characteristics of sexualized drug use of different drugs by age, among MSM^a^ from Madrid and Barcelona (%).

		**All**	**Age**			***P*** **-value**
			**<25**	**25–39**	**≥40**	
**Ever-sexualized drug use** ^**b**^	Any drug	56.0	48.2	58.7	54.9	^†^, ^¥^
	Viagra	21.9	9.7	20.3	32.5	^†^, ^¥^, ^‡^
	Poppers	44.5	37.8	47.8	41.2	^†^, ^‡^
	Cannabis	21.3	22.3	23.2	16.8	^¥^, ^‡^
	Amphetamine	6.5	4.4	7.7	5.1	^†^, ^‡^
	Cocaine	15.2	7.7	16.7	16.2	^†^, ^¥^
	Ecstasy	11.7	8.6	13.6	9.3	^†^, ^‡^
	Ketamine	7.2	6.4	8.2	5.6	^‡^
	GHB/GBL	13.4	10.0	15.7	10.5	^†^, ^‡^
	Methamphetamine	8.2	5.8	9.6	6.5	^†^, ^‡^
	Mephedrone	8.3	5.8	9.4	7.2	^†^
**Continuity of sexualized use (last month)** ^**c**^	Viagra	53.6	47.7	49.7	60.0	^‡^
	Poppers	53.3	44.4	54.3	55.4	^†^, ^¥^
	Cannabis	47.5	52.0	49.7	37.0	^¥^, ^‡^
	Amphetamine	30.5	45.0	28.1	30.8	
	Cocaine	32.6	44.1	34.9	24.2	^¥^, ^‡^
	Ecstasy	26.8	35.1	26.6	22.9	
	Ketamine	31.8	46.2	32.8	19.5	^¥^
	GHB/GBL	45.3	50.0	46.7	38.3	
	Methamphetamine	43.0	50.0	43.7	37.3	
	Mephedrone	48.3	41.7	48.4	50.9	
**First sexualized drug in his life**^**c**^^†^,^¥^,^‡^	Viagra	12.2	6.1	7.7	25.4	
	Poppers	53.6	52.8	55.5	49.6	
	Cannabis	19.6	31.1	20.4	11.9	
	Amphetamine	0.5	0.5	0.4	0.7	
	Cocaine	4.9	0.9	5.8	4.8	
	Ecstasy	2.6	2.4	2.5	3.1	
	Ketamine	0.2	0.5	0.2	0.0	
	GHB/GBL	3.1	2.4	3.9	1.7	
	Methamphetamine	1.5	1.9	1.8	0.7	
	Mephedrone	1.2	0.0	1.6	1.0	
**Drug most used as sexulized in his life**^**c**^^†^,^¥^,^‡^	Viagra	12.1	4.3	8.1	25.2	
	Poppers	55.7	58.6	57.4	50.2	
	Cannabis	17.8	27.6	18.6	11.1	
	Amphetamine	0.6	0.5	0.7	0.5	
	Cocaine	4.5	0.5	4.7	5.9	
	Ecstasy	2.0	2.9	2.0	1.7	
	Ketamine	0.2	0.5	0.2	0.0	
	GHB/GBL	2.8	2.9	3.2	1.9	
	Methamphetamine	1.9	1.0	2.2	1.7	
	Mephedrone	1.8	0.5	2.3	1.2	
**Always/most of the time for sex in last 12 months** ^**c**^	Cannabis	51.1	45.5	51.5	54.0	
	Amphetamine	52.9	60.0	47.2	68.2	
	Cocaine	46.8	35.3	43.5	58.4	
	Ecstasy	35.1	27.8	36.1	35.3	
	Ketamine	52.9	50.0	52.2	55.9	
	GHB/GBL	67.6	73.9	66.9	66.7	
	Methamphetamine	78.3	81.3	73.0	91.6	^‡^
	Mephedrone	75.4	75.0	72.0	83.3	

^a^MSM, men who have sex with men.

^b^The percentages have been calculated on the total number of participants.

^c^The percentages have been calculated on the number of participants who had consumed each substance to have sex.

† *p*-value (< 25 vs 25–39) < 0.05;

¥ *p*-value (< 25 vs ≥40) < 0.05;

‡ *p*-value (25–39 vs ≥40) < 0.05.

Continuity of use was very high; more than 50% of those who had ever used Viagra and poppers for SDU had also used them in the past month, and this proportion was 40% for cannabis, GHB, methamphetamine and mephedrone. Viagra and poppers had greater continuity among the older age group, while the other drugs generally had greater continuity among the younger group, although these differences were not statistically significant.

Poppers were the first drug consumed as SDU for 53.6% of all participants and for almost half in all three age groups, followed by Cannabis (19.6%) and Viagra (12.2%). However, there were clear differences by age. While cannabis use was more frequent among young people (31.1%), Viagra was more frequent among older people (25.4%). Less than 15% used any of the other substances as a first drug, and the percentage was substantially lower among young people (8.5%), due to the almost non-existent use of cocaine as a drug of sexualized initiation. The most commonly used SDU drugs were the same three that were most commonly used for first-time drug use, with practically identical percentages.

With the exception of ecstasy (35.1%), more than half of those who had ever used each of the SDUs had always, or most often, used such drugs specifically for that purpose in the past 12 months. This proportion was substantially higher for the three drugs most commonly associated with the chemsex phenomenon: GHB/GBL (67.6%), Mephedrone (75.4%) and methamphetamine (78.3%), especially among those aged >40, where this proportion reached 91.6%.

### Characteristics of the first episode of sexualized use

Between 23.1% and 28.0% of the participants with SDU, their first time using the three drugs considered most recreational (amphetamine, cocaine and ecstasy) had been for sex, with no differences by age. Ketamine (41.9%) and GBH (56.6%) were in intermediate positions, while methamphetamine and mephedrone were used by about 70%. In addition, SDU was observed to take place quite early, as less than half of those who had SDU did so after having used recreational drugs for other purposes for at least 5 days. In the case of methamphetamine and mephedrone less than 16% did so. First-time sexualized use increased significantly with age for mephedrone (Table 3).

**Table 3:** Characteristics of the first episode of sexualized use for different drugs by age among MSM^a^ from Madrid and Barcelona.

			**All**	**Age**			***P*** **-value**
				**<25**	**25–39**	**≥40**	
**Purpose of the first use** ^**b**^	**Sexualized use in the first use**	Amphetamine	28.0	25.0	28.3	28.2	
		Cocaine	23.6	23.5	24.2	22.4	
		Ecstasy	23.1	13.5	24.6	23.6	
		Ketamine	41.9	42.9	40.3	46.3	
		GHB/GBL	56.6	65.9	52.5	64.6	
		Methamphetamine	69.6	61.5	70.6	70.6	
		Mephedrone	72.2	58.3	69.5	85.7	^†^, ^¥^
	**Have used** ** <5 days for other purpose before first sexualized use**	Amphetamine	26.3	25.0	27.6	23.1	
		Cocaine	30.1	26.5	30.8	29.6	
		Ecstasy	27.9	35.1	28.1	23.6	
		Ketamine	23.6	21.4	22.4	29.3	
		GHB/GBL	19.2	15.9	21.2	14.6	
		Methamphetamine	16.0	19.2	15.0	17.6	
		Mephedrone	12.0	8.3	13.6	8.9	^†^, ^¥^
	**Have used** **>5 days for other purpose before first sexualized use**	Amphetamine	45.7	50.0	44.1	48.7	
		Cocaine	46.3	50.0	45.1	48.0	
		Ecstasy	48.9	51.4	47.3	52.8	
		Ketamine	34.5	35.7	37.3	24.4	
		GHB/GBL	24.2	18.2	26.3	20.7	
		Methamphetamine	14.3	19.2	14.4	11.8	
		Mephedrone	15.8	33.3	16.9	5.4	^†^, ^¥^
**Intentionally for sex** ^**b**^		Amphetamine	23.8	20.0	21.4	33.3	
		Cocaine	27.1	23.5	25.0	32.5	
		Ecstasy	14.6	16.2	14.5	14.1	
		Ketamine	36.6	28.6	36.8	41.5	
		GHB/GBL	61.9	54.5	60.0	72.0	^¥^
		Methamphetamine	71.5	65.4	72.3	72.0	
		Mephedrone	66.8	69.6	63.9	73.7	
**Mode of drug acquisition** ^**b**^	**Had got the drug before**	Amphetamine	56.2	65.0	57.1	48.7	
		Cocaine	48.2	52.9	51.3	40.0	
		Ecstasy	63.0	75.7	62.7	57.1	
		Ketamine	39.8	53.6	41.7	24.4	^¥^, ^‡^
		GHB/GBL	30.3	39.5	28.1	32.1	
		Methamphetamine	16.2	19.2	15.2	18.0	^†^
		Mephedrone	24.7	37.5	24.7	19.3	
	**Bought it where he had sex**	Amphetamine	2.7	5.0	2.4	2.6	
		Cocaine	2.8	5.9	2.2	3.2	
		Ecstasy	5.1	2.7	5.8	4.3	
		Ketamine	3.5	7.1	1.5	7.3	^¥^, ^‡^
		GHB/GBL	4.7	7.0	3.9	6.2	
		Methamphetamine	4.7	15.4	3.8	2.0	^†^
		Mephedrone	8.5	8.3	10.4	3.5	
	**Was given free where he had sex**	Amphetamine	41.1	30.0	40.5	48.7	
		Cocaine	49.1	41.2	46.5	56.8	
		Ecstasy	31.9	21.6	31.6	38.6	
		Ketamine	56.7	39.3	56.8	68.3	^¥^, ^‡^
		GHB/GBL	65.0	53.5	68.0	61.7	
		Methamphetamine	79.1	65.4	81.0	80.0	^†^
		Mephedrone	66.8	54.2	64.9	77.2	
**Had sex only with steady partner** ^**b**^		Amphetamine	14.5	5.0	16.5	12.8	
		Cocaine	17.7	20.6	14.8	23.6	^‡^
		Ecstasy	18.4	16.2	17.0	23.9	
		Ketamine	12.0	14.3	10.7	14.6	
		GHB/GBL	9.7	9.1	9.3	11.1	
		Methamphetamine	6.8	7.7	7.0	6.0	
		Mephedrone	8.1	16.7	7.1	7.1	
**Did not used a condom with any partner** ^**c**^		Amphetamine	34.6	26.3	33.0	44.1	
		Cocaine	27.2	37.0	27.5	23.4	
		Ecstasy	26.2	22.6	25.3	31.5	
		Ketamine	45.5	37.5	47.0	45.7	
		GHB/GBL	40.8	37.5	40.2	44.4	
		Methamphetamine	56.9	58.3	53.7	66.0	
		Mephedrone	50.2	45.0	49.7	53.8	

^a^MSM, men who have sex with men.

b The percentages have been calculated on the number of participants who had consumed each substance to have sex.

c The percentages have been calculated on the number of participants who had consumed each substance for SDU with occasional partners (regardless of whether or not he also had sex with his steady partner)

† *p*-value (< 25; 25–39) < 0.05;

¥ *p*-value (< 25; ≥40) < 0.05;

‡ *p*-value (25–39; ≥40) < 0.05.

If we take as a reference the total number of users of a substance and not only those who have used it for SDU, the percentage for whom SDU was the first experience, the gateway to use, was logically lower: 40.8% for Mephedrone, 35.7% for methamphetamine, 34.2% for GHB/GBL and 17.0% for ketamine, while for recreational drugs it was around 10%.

Most of the participants did not intentionally use for sex the three recreational drugs studied on their first sexualized use (irrespective of whether it had been consumed before for recreational purposes or not), even if they ultimately had sex under the effect of these substances. In the case of ecstasy, only 14.5% used it intentionally for sex. However, the situation was reversed with the 3-chems: 61.9% used GHB intentionally for sex, as did 71.5% in the case of methamphetamine and 66.8% in the case of mephedrone. The mode of drug acquisition in participants' first SDU had a similar pattern to that of intentionality for sex: in the case of recreational drugs, half or more of the subjects had acquired them prior to having sex, while in the case of the 3-chems, more than 65% obtained them for free at the place where they had sex. Only ketamine and methamphetamine showed differences by age, with young people obtaining them free in the lowest proportion. Mephedrone was the drug that the highest percentage (8.5%) bought at the place where they had sex.

For participants' first SDU, only 15–20% using recreational drugs and less than 10% using three-chemsex drugs had sex only with their steady partner; the vast majority had sex with casual partners or both. Among those who had sex with one or more occasional partners, between 26.2% and 34.6% of recreational drug users did not use a condom with anyone, while the percentage was over 40% for 4-chems.

### Correlates of sexualized use in the first use

Supplementary Table 4 of the Annex shows the crude results of the analysis. In the multivariate model (Table 4), sexualized use in the first use, in the case of party drugs, was associated with being tested in Barcelona and having been born in Latin America, whereas chemsex drugs were associated with being recruited in STI diagnostic center. The other associated factors were common for both kind of drugs. The most strongly associated factor was having been penetrated by more than 20 men in one's lifetime, in which aPRs were around five for both kind of drugs, followed by ever injected drugs [(aPR: 4.0; 95% CI:3.1–5.0, for chemsex drugs) and (aPR 2.0; 95% CI:1.2–3.3, for party drugs)]. The remaining factors (met the largest number of partners at discos/bars-saunas-private parties, ever having been paid for sex, ever injected steroids and ever diagnosed with an STI) showed similar aPRs in both populations, of between 1.4 and 1.5.

**Table 4:** Multivariate regression analysis of associated factors with sexualized use in the first use for recreational drugs and for chemsex drugs.

	**Sexualized use in the first use for recreational drugs** ^**1**^		**Sexualized use in the first use for chemsex drugs** ^**2**^	
	aPR^*^	CI 95%^**^	**aPR**	**CI 95%**
**City of testing**
Madrid	1.0			
Barcelona	**1.5**	1.1–2.0		
**Kind of testing program**
Comunity program			1.0	
STI diagnostic center			**1.3**	1.1–1.6
**Country of birth**
Spain	1.0			
Latin America	**1.6**	1.2–2.2		
Others	1.3	0.8–2.0		
**Lives sex life with men**
Not openly	1.0		1.0	
Openly	1.3	0.9–1.8	**1.3**	1.0–1.6
**Place where the largest number of partner were found**
Others	1.0		1.0	
Discos/bars-saunas-private parties	**1.8**	1.3–2.4	**1.7**	1.4–2.1
**Number of men who had penetrated you (ever)**
None-one	1.0		1.0	
2–20	**4.3**	1.4–13.5	**3.2**	1.4–7.2
>20	**4.9**	1.5–15.4	**5.0**	2.2–11.2
**Ever been paid for sex**
No	1.0		1.0	
Yes	**1.4**	1.1–1.9	**1.5**	1.2–1.8
**Ever injected drugs**
No	1.0		1.0	
Yes	**2.0**	1.2–3.3	**4.0**	3.1–5.0
**Ever injected steroids**
No	1.0		1.0	
Yes	**1.6**	1.1–2.5	**1.5**	1.2–2.0
**Ever diagnosed with an STI**
No	1.0		1.0	
Yes	**1.5**	1.1–2.0	**1.8**	1.4–2.3

1 Amphetamine, cocaine or ecstasy.

2 Ketamine, GHB/GBL, methamphetamine or mephedrone.

Bold values indicate that the adjusted prevalence ratio is significant.

* Adjusted prevalence ratio.

** 95% confidence interval.

## Discussion

### Main findings

To our knowledge, this is the first study that jointly analyzes drug use for any purpose and for sex (SDU) among MSM. It is also the first study to analyze to what extend SDU has served as the gateway to drug use, the first consumption experience for different drugs, and various characteristics of the first sexualized consumption of these drugs.

Thus, this study represents the first time it has been possible to provide empirical evidence that, among MSM, SDU is one of the most prevalent motivations or contexts for drug consumption, especially Viagra, poppers, cocaine and chemsex drugs, for which the prevalence of SDU is close to or above 50% of the prevalence of use for any purpose, both during one's lifetime and in the past 12 months. In contrast, it has been confirmed that hallucinogens, sedatives and opiates are not generally used for SDU. We have used the word “gateway,” referring only to the first experience with different drugs, without any related meaning with the classical gateway drug effect or stepping-stone theory (37). The age group with the lowest lifetime prevalences of SDU for almost all drugs was people under 25, while those aged 25–39 have the highest prevalence. While it is clear that the lifetime prevalence of younger people may be more likely to increase, the data suggest that the phenomenon of increased sexualization of use has perhaps begun years ago. An analysis carried out by the 2012 EMIS Study (5) showed a decline by age in recent 4-chem prevalence.

This study shows, also for the first time, that SDU serves as the first experience of drug use; it is the gateway to consumption for more than one in three users of methamphetamine, mephedrone and GHB. This is also the case for about 1 in 10 users of the recreational drugs: cocaine, ecstasy and amphetamine. Among MSM with SDU, these percentages double or triple, depending on the drug. In addition to those who consumed directly through SDU, another significant percentage of individuals entered SDU very early (having used less than five times for other purposes). The drugs most commonly used during the first episode of SDU were the three that were most prevalent for SDU: poppers (in more than half of the cases), cannabis (one in five), and Viagra (just over 1 in 10), although the order of these last two substances was reversed in the case of those over age 40. This study also suggests that the context of the first sexualized use is very different for recreational drugs compared to chemsex drugs. The former are obtained in greater proportion before having sex, while chemsex drugs are obtained in the same place where sex is carried out, mostly provided as gifts, thus facilitating entry into consumption. It is also clear that this consumption usually takes place in contexts of sexual risky behaviors, since it is rare to have sex only with a stable partner, rather it is common to have sex with occasional partners and with more than one person for a high percentage of individuals, especially in the case of chemsex drugs. In addition, more than one in four (for recreational drugs) and nearly half or more (for chemsex drugs) do not use a condom with any sexual partner. This suggests that, from the beginning, there is a “risk ladder” in terms of risky behavior (10) depending on the drug type.

Poppers, cannabis and Viagra were the substances that participants had used most often in a sexualized way in their lifetime. It should be noted that among those who have ever used a substance for SDU, this type of use becomes the predominant context for their use. This is because, with the exception of ecstasy, more than half had used the substance always or most of the time for sex in the last year. There are currently no other studies that corroborate this finding, because other studies, such as the EMIS study, have not used this approach. Rather, they have focused analysis on the percentage of sober sex (35). It is also worth noting that more than half of those whose first use of recreational drugs was for SDU also used chemsex drugs. However, less than a third of those who used chemsex drugs for SDU the first time also used recreational drugs. This finding can be explained by the fact that, as we have pointed out, entry into chemsex use for the purpose of SDU is much more frequent. In addition, it is possible that there is a time sequence that begins with SDU use of recreational drugs, however we cannot confirm this, because we did not ask about the age of onset.

The study also shows, for the first time, that drugs that are assumed to always be consumed for sex (Viagra) or almost always (poppers), are actually consumed on quite a few occasions without sex. In fact, in this study we did not ask whether the first use of these two substances was sexualized or not, since we assumed that it was practically always sexualized. This phenomenon is even more relevant in the case of chemsex drugs, since nearly half had not used them for this purpose. However, many studies analyze the use of these substances as if all users were using them for sex (5). It is quite possible that poppers, and especially Viagra, are often consumed with the thought that sexual intercourse will most likely take place under their effect, but then this does not happen. On the other hand, recreational drugs are most likely taken in a recreational setting where sex is often involved, but the drugs are not intentionally taken for that purpose. In this study the percentage who had intentionally taken them for sex at the first SDU was less than 30%. However, all three chem drugs are taken intentionally for sex on most occasions by those who have used them for SDU, most likely in the context of organized chemsex sessions.

Being recruited in Barcelona and being born in Latin America was associated with having consumed any of the three recreational drugs analyzed for SDU, which could be explained by cultural differences that promote greater sexualization of these drugs with respect to those born in Spain and residing in Madrid. The association between sexualized initiation of chemsex drugs and being recruited in STI diagnostic centers is most likely due to the fact that these centers serve a population with more risky behaviors. All other correlates were common for recreational and chemsex drugs. Those whose sexual behavior is more open had a higher risk, as well as those who find their partners mainly in discos, bars and private parties. This finding is surprising because the chemsex phenomenon has always been related to the ease of contact through dating apps, which was in fact the most frequent way of obtaining partners among the participants. It is possibly due to the fact that SDU at first use occurs among those who frequent such environments for possibly all recreational drugs, and also for some chemsex drugs. However, the correlates with the strongest association were common for recreational and chemsex drugs, and all were indicators of risk behaviors. Having been penetrated by more than 20 men in life, ever having injected drugs and ever having been diagnosed with an STI (especially for chemsex drugs) are worth highlighting. This association with risk behaviors was to be expected, especially for chemsex drugs. Since it is already known that such an association exists for those who have practiced SDU (10, 18, 34, 38), it is logical that it would be found among people who start using those drugs for sex. That the strongest correlates are common to both drugs seems logical, since, as we have pointed out, a large percentage began consumption as SDU for both types of drugs.

### Limitations

As in most studies in MSM, this study employed convenience sampling, here MSM accessing HIV testing. A sample of significant size was recruited, and two programs with very different client recruitment characteristics (one health center and one community program) were chosen in each city in order to increase the sample's heterogeneity and representativeness. Still, caution should be exercised in generalizing results among MSM, because convenience samples tend to sample higher-risk MSM than do general population surveys (39, 40). However, this bias could be partly compensated for the fact that HIV positive men were not included, and it is known that this group presents higher levels of drug use than those who are HIV-negative (5, 41, 42). Secondly, the participants came only from the Madrid and Barcelona areas. These are the two most populated areas in Spain and have higher prevalences of MSM, because they attract MSM from other parts of Spain and abroad. Previous studies have shown that they are among the European cities with the highest SDU prevalences (5, 34). It is probable that their prevalences are higher than other less populated areas of Spain, as has also been described in other countries (43). Thirdly, the presence of social desirability bias could be thought to compromise the validity of socially disapproved behaviors. However, the collection of information through an anonymous, self-administered questionnaire tends to reduce such bias compared to telephone or face-to-face interviews, since it preserves to a greater extent the privacy of responses to questions of a more sensitive nature. Finally, in order to make it easier for participants to answer the questionnaire, the same time period was used to define sexualized use (6 h). This may have led to some overestimation of SDU for some substances with shorter half-lives (such as poppers or mephedrone), although it is common for these substances to be taken repeatedly, before or during sex.

### Conclusions and policy and research implications

Prevalence of SDU was more than half of the prevalence of drug use for any purpose. Thus, SDU acts as the gateway to use for many drugs in an important proportion of users, who frequently have their first consumption experience using free drugs while having condomless, anal sex with occasional and multiple partners. In addition, among 50% or more of those who had SDU, this context of consumption was the predominant one, accounting for drug use always or most of the time during the past year. These circumstances were much more common for chemsex than for recreational drugs. It is highly probable that the popularization of some substances used primarily for SDU (especially methamphetamine and mephedrone) will facilitate their use for recreational purposes in this same population. MSM could serve as a bridge, bringing these drugs to the general population, where their use is currently very low. Beginning substance use directly through SDU was found to be associated with a higher level of both sexual and injection risk behaviors, suggesting that we are dealing with a population that is particularly vulnerable to infectious diseases transmitted by these routes. However, neither level of education nor economic status was found to have an association, while openly living one's sexual life as gay was.

In this context, it is important to reinforce the informative, preventive and harm reduction initiatives that are already being implemented in various countries and that take into account the interventions suggested by MSM themselves (44–47). It is important not to forget the challenge posed by the likely existence of culture that is counter to public health. As a recent study in Australia has shown, this culture has been found to be underpinned by forms of “sex-based sociality,” which gives primacy to the priorities and practices of gay and bisexual men (48). It would also be useful to have information similar to that collected here for the general population, since the prevalence of SDU for the different substances is unknown, as is whether SDU acts as a privileged gateway to consumption.

## Data availability statement

The original contributions presented in the study are included in the article/Supplementary materials, further inquiries can be directed to the corresponding author/s.

## The methysos project group

María del Carmen Burgos and César Pérez Romero (Instituto de Salud Carlos III, Madrid); José Antonio San Juan Bueno (Asociación Pink Peace, Madrid); Francisca Román Urrestarazu, Jesus E. Ospina, and Miguel Alarcón Gutiérrez (Agència de Salut Pública de Barcelona, Barcelona); Jorge del Romero, Carmen Rodríguez, Sonsoles del Corral Del Campo, Natividad Jerez Zamora, Marta Ruiz Fernández, and Montserrat González Polo (Centro Sanitario Sandoval, Madrid); María Jesús Barbera Gracia, Luis López Pérez, Claudia Broto Cortes, and Julio Morais Martin (UITS Drassanes, Barcelona).

## Author contributions

LF, PG, and M-JB conceived, designed, and supervised the study. J-MG and DD organized the recruitment of participants. J-MG, MD, DD, OA, and JG-P contributed to the recruitment of subject and data collection. J-MG, JH, MD, and DD performed the main analyzes. J-MG, JH, LF, PG, and M-JB wrote the manuscript. JH, LF, and M-JB were responsible for drafting and critical revisions of the manuscript. J-MG, JH, MD, LF, DD, OA, JG-P, PG, M-JB, and the additional members of the Methysos Project Group made substantive contributions to the current article. All authors read and approved the final manuscript.

## Funding

This study was supported by the Delegación del Gobierno para el Plan Nacional sobre Drogas (2019I017).

## Conflict of interest

The authors declare that the research was conducted in the absence of any commercial or financial relationships that could be construed as a potential conflict of interest.

## Publisher's note

All claims expressed in this article are solely those of the authors and do not necessarily represent those of their affiliated organizations, or those of the publisher, the editors and the reviewers. Any product that may be evaluated in this article, or claim that may be made by its manufacturer, is not guaranteed or endorsed by the publisher.

## Abbreviations

aPR: adjuted prevalence ratio
CI: confidence interval
cPR: crude prevalence ratio
HCV: hepatitis C virus
MSM: men who have sex with men
SDU: sexualized drug use
STI: sexually transmitted infection
UK: United Kingdom.
