# Supplementary material for: Sexualized drug use among men who have sex with men in Madrid and Barcelona: The gateway to new drug use?
Source: Front Public Health. 2022 Nov 15;10:997730. doi: 10.3389/fpubh.2022.997730 (PMC9705339; doi:10.3389/fpubh.2022.997730)
Supplement: Supplementary file 1 [file Data_Sheet_1.PDF]

ANNEX: Table 1. Original variables and categories from table 1, as they were collected in the questionnaire and their corresponding variables and categories as they have been employed in the analysis (I).

Original form of variables and categories as used in the questionnaire	Variables and categories employed in the analysis
**Recruitment**	
**City of testing**	**City of testing**
Madrid	Madrid
Barcelona	Barcelona
**Kind of testing programme**	**Kind of testing program**
Pink Peace programme	Community program
Agencia de Salud Pública de Barcelona	
Centro Sanitario Sandoval	STI diagnostic center
UITS Drassanes	
**Sociodemographics**	
**Age (years)**	**Age (years)**
Continuous variable	<25
	25-39
	≥40
**Country of birth**	**Country of birth**
Open answer	Spain
	Latin-America
	Others
**Size of city of residence (last 12 months)**	**Size of city of residence (last 12 months)**
Less than 10 thousand	≤ 100.000
More than 10 thousand	
More than 50 thousand	
More than 100 thousand	100.000 - 1 million
More than 500 thousand	
More than a million	> 1 million
**Level of education**	**Level of education**
None or primary education (the level that should be finished at 12 years of age)	Up to upper secondary
Lower secondary or second stage of Basic education: designed to complete Basic education, usually following a more subject-oriented pattern (the level that should be finished at 16 years of age)	
(upper) secondary education: more specialized education typically beginning at age 15 or 16 years and/or the end of compulsory education (the level that should be finished at 18 years of age)	Post secondary
Post-secondary, non-tertiary education: captures programs that straddle the Boundary between upper- and post-secondary education from an International point of view as pre-university courses of short vocational programs (the level that should be finished at 20 years of age)	
Higher education university education: specific vocational training, first and second university degrees, Bachelor, Master degree, Doctorate (PhD)	University
**Employment status (last 12 months)**	**Employment status (last 12 months)**
Employed full-time	Employed
Employed part-time	
Self-employed	
Unemployed (with or without subsidy)	Unemployed
Student	Others
Long-term sick leave/ medically retired	
Retired	
**Economic situation (last 12 months)**	**Economic situation (last 12 months)**
Very comfortable	Comfortable/ It is OK
Comfortable, it is all right	
It is tight, I need to be careful	Tight
I make ends meet with difficulties	Difficult/Very difficult
I am unable to make ends meet without debt.	
**Cohabitation (last 12 months)**	**Cohabitation (last 12 months)**
Alone	Alone
With some people	With some people

ANNEX: Table 1. Original variables and categories from table 1, as they were collected in the questionnaire and their corresponding variables and categories as they have been employed in the analysis (II).

Original form of variables and categories as used in the questionnaire	Variables and categories employed in the analysis
**Sexual behavior**	
**You have ever had sex with….**	**Gender of sex partners (ever)**
Only with men	Only men
More often with men, but at least once with a woman	Men & women
About equally often with men and with women	
More often with women, but at least once with a man	
**Age at first sexual intercourse with another men (years)**	**Age at first sexual intercourse with another man (years)**
Continuous variable	≤ 15
	16-20
	21-24
	≥ 25
**You live sex-life with men…**	**Lives sex-life with men…**
Openly	Openly
Discreetly	Not Openly
Hidden	
In total secrecy	
**Place where you found the largest number of partners**	**Place where the largest number of partners were found**
Discotheques, clubs and gay bars	Discos/clubs/bars
Saunas	Saunas
A sex party in a private home	Private parties
Apps	Apps/websites
Internet	
Parks, public restrooms, and other places for flirting or "cruising"	Cruising places
Sex Clubs	Others/no search
Dark Rooms, sex shops	
Other: specify	
**Place where you found the largest number of partners**	**Place where the largest number of partners were found**
A sex party in a private home	Discos/bars-saunas-private parties
Discotheques, clubs and gay bars	
Saunas	
Apps	Others
Internet	
Parks, public restrooms, and other places for flirting or "cruising"	
Sex Clubs	
Dark Rooms, sex shops	
Other: specify	
**Risk behavior**	
**Number of men who have penetrated you (ever)**	**Number of men who have penetrated you (ever)**
None	None-One
One	
Less than 5	2-20
Less than 10	
Less than 20	
Less than 50	> 20
Less than 100	
Less than 200	
More than 200	
**Number of men who have penetrated you (last 12 months)**	**Number of men who have penetrated you (last 12 months)**
None	None-One
One	
Less than 5	1-5
Less than 10	> 5
Less than 20	
Less than 50	
Less than 100	
Less than 200	
More than 200	
**Number of men who have ever paid you for sex**	**Ever been paid for sex**
None	No
One	Yes
Less than 5	
Less than 10	
Less than 20	
Less than 50	
Less than 100	
More than 100	

ANNEX: Table 1. Original variables and categories from table 1, as they were collected in the questionnaire and their corresponding variables and categories as they have been employed in the analysis (III).

Original form of variables and categories as used in the questionnaire	Variables and categories employed in the analysis
**Risk behavior**	
**Number of men you have ever paid for sex**	**Ever paid for sex**
None	No
One	Yes
Less than 5	
Less than 10	
Less than 20	
Less than 50	
Less than 100	
More than 100	
**Ever injected drugs**	**Ever injected drugs**
No	No
Yes	Yes
**Ever injected steroids**	**Ever injected steroids**
No	No
Yes	Yes
**History of HIV and other STI testing**	
**Number of previous HIV tests / Occurrence of last HIV testing episode**	**Time since last HIV test**
Once; 2 times; 3 to 5 times; 6 to 9 times; 10 to 15 times; 16 to 20 times; More than 20 times → **When was the last time?** In the last month; In the last 6 months	< 6 months
Once; 2 times; 3 to 5 times; 6 to 9 times; 10 to 15 times; 16 to 20 times; More than 20 times → **When was the last time?** In the last 12 months; In the last 5 years; More than 5 years ago	> 6 months
Never	Never tested before
**HIV diagnosis in the recruitment consultation**	**HIV diagnosis in the recruitment consultation**
Based on the result of the HIV test	No
	Yes
**STI diagnosis (ever)**	**Ever diagnosied with an STI**
None	No
Syphilis	Yes
Gonorrhoea	
Chlamydia	
Lymphogranuloma venereum	
Anal or genital warts	
Genital or anal herpes	

ANNEX: Table 1. Original variables and categories from table 2, as they were collected in the questionnaire and their corresponding variables and categories as they have been employed in the analysis.

Original form of variables and categories as used in the questionnaire		Variables and categories employed in the analysis
**Ever-sexualized drug use**		**Ever-sexualized drug use**
		Any drug
Viagra®, Cialis®, Levitra® or others that help erection		Viagra
Poppers		Poppers
Cannabis or synthetic cannabinoids (marijuana, hashish, chocolate, joints, grass, synthetic marijuana, spice, K2)		Cannabis
Amphetamine (speed)		Amphetamine
Powdered or crack cocaine (theme, lighthouse, base, basuco, etc.)		Cocaine
Ecstasy or MDMA in its pill form (pills, lollipops) or in its crystalline or powder form (M, crystal)		Ecstasy
Ketamine (K, keta, kei)		Ketamine
GHB/GBL (G, canister, liquid ecstasy)		GHB/GBL
Methamphetamine (bathtub, crystal meth, T)		Methamphetamine
Mephedrone (mefe) or other different synthetic stimulants (bath salts, methoxetamine/MXE, methylone/3MMC, methylethcathinone/4MEC, fluoroamphetamine/light ecstasy/4FA)		Mephedrone

For each drug used for sexuaulized drug use

**How long has it been since you last used this drugs for sex?**		**Continuity of sexualized use (last month)**
In the last month		
In the last 6 months		
In the last 12 months		
In the last 5 years		
More than 5 years ago		

**First sexualized drug in his life**		**First sexualized drug in his life**
(list of drugs)		

**Drug most used as sexulized in his life**		**Drug most used as sexulized in his life**
(list of drugs)		
**In the LAST 12 MONTHS, you have used this drug**		**Always/most of the time for sex in last 12 months**
Always just before or during sex		
Most of the time just before or during sex		
Half the time just before or during sex.		
Rarely in relation to sex		

ANNEX: Table 1. Original variables and categories from table 3, as they were collected in the questionnaire and their corresponding variables and categories as they have been employed in the analysis.

Original form of variables and categories as used in the questionnaire		Variables and categories employed in the analysis
**For each drug used for sexuaulized drug use**		
**The FIRST TIME you used it in the 6 hours before or during anal sex**		**Purpose of the first use**
It was the first time I had tried the substance		Sexualized use in the first use
I had already taken it before I used it for sex		Have used <5 days for other purpose before first sexualized use
**How many days had you used it before using it for sex?**		
One		
Less than 5		
Less than 10		Have used >5 days for other purpose before first sexualized use
Less than 20		
Less than 50		
Less than 100		
More than 100		
**The first time you used this drug for anal sex ...**		**Intentionally for sex**
I took it so that it would have an effect during sex		
I didn't take it thinking about having sex, but I had sex while under its effect		
**Mode of drug acquisition**		**Mode of drug acquisition**
He had got the drug before		He had got the drug before
Was bought where he had sex		Was bought where he had sex
Was given free where he had sex		Was given free where he had sex
**Had sex with**		**Had sex only with steady partner**
Only the steady partner		
The steady partner and one casual partner		
The steady partner and two or more casual partners		
One casual partner		
Two or more casual partners		
**Used a condom with:**		
All the partners		
Casual partners but not with the steady partner		
Any partner		**Did not used a condom with any partner**
