# Supplementary figures and images for: Sexualized drug use among men who have sex with men in Madrid and Barcelona: The gateway to new drug use?
Source: Front Public Health. 2022 Nov 15;10:997730. doi: 10.3389/fpubh.2022.997730 (PMC9705339; doi:10.3389/fpubh.2022.997730)

**Annex: Figure 1.** Flow chart of recruitment procedures

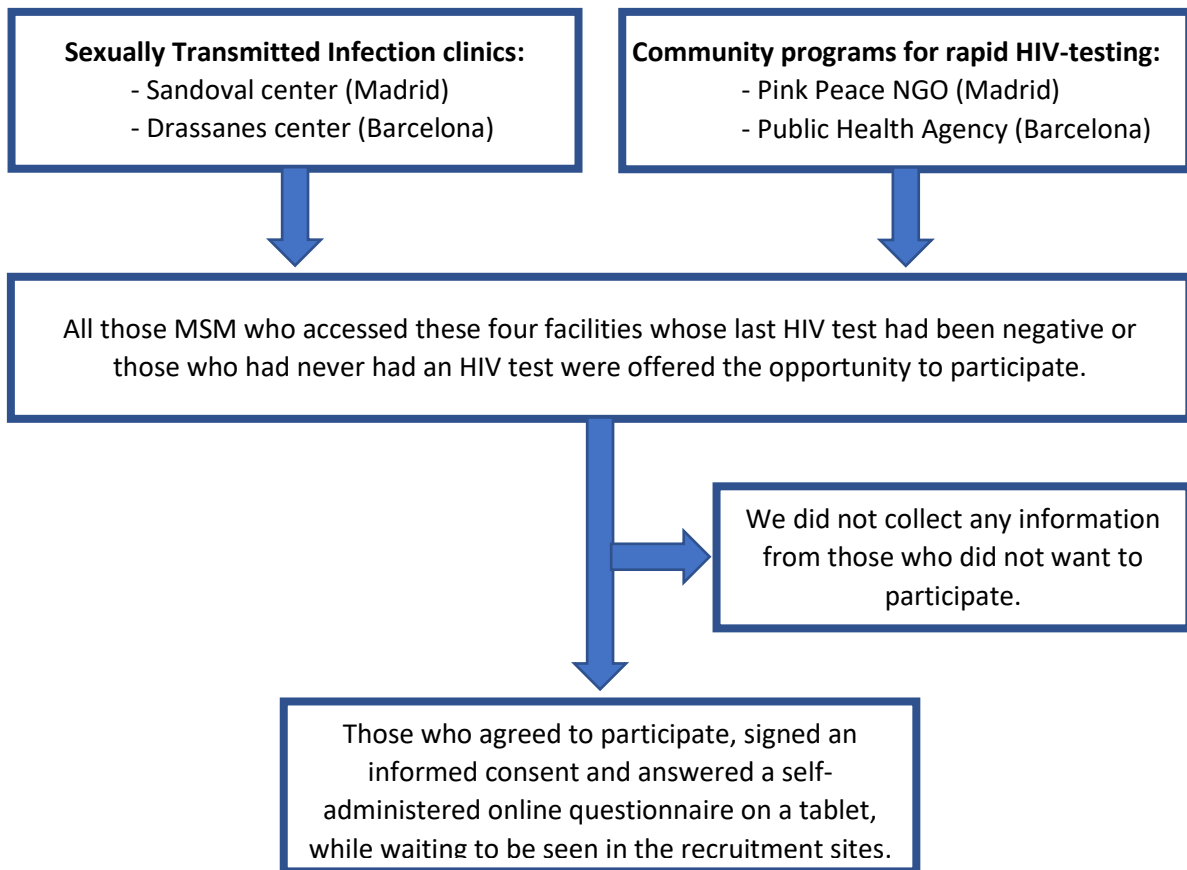

Supplement: Supplementary file 2 [file Data_Sheet_2.PDF]
