# Supplementary material for: Sexualized drug use among men who have sex with men in Madrid and Barcelona: The gateway to new drug use?
Source: Front Public Health. 2022 Nov 15;10:997730. doi: 10.3389/fpubh.2022.997730 (PMC9705339; doi:10.3389/fpubh.2022.997730)
Supplement: Supplementary file 3 [file Data_Sheet_3.PDF]

ANNEX: Table 2. Wording of the different psychoactive drugs included in the analysis.

Psychoactive drug	Wording in the questionnaire
Viagra	Viagra®, Cialis®, Levitra® or other erection aids
Poppers	Poppers
Cannabis	Cannabis or synthetic cannabinoids (marijuana, hashish, chocolate, joints, grass, synthetic marijuana, spice, K2)
Amphetamine	Amphetamine (speed)
Cocaine	Powdered or crack cocaine (theme, lighthouse, base, basuco, etc.)
Ecstasy	Ecstasy or MDMA in its pill form (pills, lollipops) or in its crystalline or powder form (M, crystal)
Ketamine	Ketamine (K, keta, kei)
GHB/GBL	GHB/GBL (G, canister, liquid ecstasy)
Methamphetamine	Methamphetamine (bathtub, crystal meth, T)
Mephedrone	Mephedrone (mefe) or other different synthetic stimulants (bath salts, methoxetamine/MXE, methylone/3MMC, methylethcathinone/4MEC, fluoroamphetamine/light ecstasy/4FA)

ANNEX: Table 3. Summary of terms used and drugs included in each term.

Terms used	Drugs included
Chemsex drugs, 4-chems	Methamphetamine, Mephedrone, GHB/GBL and Ketamine
3-chems	Methamphetamine, Mephedrone and GHB/GBL
2-chems	Methamphetamine and Mephedrone
Party drugs, Recreational drugs	Amphetamine, Cocaine and Ecstasy
