# Supplementary material for: Sexualized drug use among men who have sex with men in Madrid and Barcelona: The gateway to new drug use?
Source: Front Public Health. 2022 Nov 15;10:997730. doi: 10.3389/fpubh.2022.997730 (PMC9705339; doi:10.3389/fpubh.2022.997730)
Supplement: Supplementary file 4 [file Data_Sheet_4.PDF]

ANNEX: Table 4 Regression poisson analysis crude of associated factors with sexualized use in the first use for recreational drugs and for chemsex drugs.

	Sexualized use in the first use for recreational drugs[1]		Sexualized use in the first use for chemsex drugs[2]	
	cPR*	CI 95%**	cPR*	CI 95%**
		**Recruitment**		
**City of testing**				
Madrid	1,0		1,0	
Barcelona	**1,5**	1.1-2.0	1,1	0.9-1.4
**Kind of testing program**				
Comunity program	1,0		1,0	
STI diagnostic center	1,2	0.9-1.6	**1,6**	1.3-1.9
		**Sociodemographics**		
**Age (years)**				
<25	1,0		1,0	
25-39	**2,1**	1.2-3.5	**1,5**	1.1-2.2
≥40	1,6	0.9-2.9	1,3	0.9-1.8
**Country of birth**				
Spain	1,0		1,0	
Latin America	**2,0**	1.4-2.7	1,2	1.0-1.6
Others	**1,6**	1.0-2.5	1,1	0.8-1.5
**Size of city of residence (last 12 months)**				
< 1 million	1,0		1,0	
≥ 1 million	0,9	0.7-1.3	1,2	1.0-1.6
**Level of education**				
University	1,0		1,0	
Not university	1,1	0.8-1.4	1,2	1.0-1.5
**Employment status (last 12 months)*** **				
Employed	1,1	0.5-2.4	**0,6**	0.4-0.8
Unemployed	1,0		1,0	
Others	0,6	0.2-1.6	**0,3**	0.2-0.5
**Economic situation (last 12 months)**				
Comfortable/ It is OK	1,0		1,0	
Tight/Dificult/Very difficult	1,0	0.8-1.4	1,0	0.8-1.3
**Cohabitation (last 12 months)*** **				
Alone	1,0		1,0	
With some people	1,0	0.7-1.6	1,2	0.9-1.6
		**Sexual behavior**		
**Gender of sex partners (ever)**				
Men & women	1,0		1,0	
Only men	0,9	0.7-1.2	**0,8**	0.6-1.0
**Age at first sexual intercourse with another men (years)**				
< 16	**1,5**	1.0-2.1	**1,4**	1.1-1.8
≥ 16	1,0		1,0	
**Lives sex life wiht men…**				
Not Openly	1,0		1,0	
Openly	**1,6**	1.2-2.2	**1,8**	1.4-2.3
**Place where the largest number of partnes were found**				
Discos/bars-saunas-private parties	**2,0**	1.5-2.6	**2,1**	1.7-2.6
Others	1,0		1,0	
		**Risk behavior**		
**Number of men who had penetrated you (ever)**				
None-One	1,0		1,0	
2-20	**5,5**	1.7-17.5	**4,1**	1.8-9.4
>20	**8,7**	2.7-27.2	**9,7**	4.3-21.8
**Ever been paid for sex**				
No	1,0		1,0	
Yes	**2,0**	1.5-2.7	**2,1**	1.7-2.7
**Ever paid for sex**				
No	1,0		1,0	
Yes	**1,4**	1.0-2.0	1,2	0.9-1.6
**Ever injected drugs**				
No	1,0		1,0	
Yes	**3,1**	1.8-5.5	**6,9**	5.1-9.2
**Ever injected steroids**				
No	1,0		1,0	
Yes	**2,5**	1.6-3.8	**2,8**	2.1-3.7
		**History of HIV and other STI testing**		
**Time since last HIV test**				
Never tested before	1,0		1,0	
< 6 months	**3,5**	1.3-9.6	**3,9**	1.6-8.4
> 6 months	2,3	0.8-6.3	**2,5**	1.2-5.3
**HIV diagnosis in the recruitmen consultation**				
No	1,0		1,0	
Yes	0,6	0.1-2.3	1,5	0.8-2.8
**Ever diagnosied with an STI**				
No	1,0		1,0	
Yes	**2,0**	1.42.7	**2,8**	2.2-3.6

[1] Amphetamine, cocaine or ecstasy
[2] Ketamine, GHB/GBL, methamphetamine or mephedrone
* Crude Prevalence Ratio
** 95% Confidence Interval
*** These questions were not included in Barcelona
